# Implications for blinding in clinical trials with THC-containing cannabinoids based on the CANNA-TICS trial

**DOI:** 10.3389/fnins.2022.793703

**Published:** 2022-09-21

**Authors:** Kirsten R. Müller-Vahl, Ewgeni Jakubovski, Carolin Fremer, Martina Lenz-Ziegenbein, Anika Großhennig, Carolin Klages, Armin Koch, Martina Haas, Anna Pisarenko

**Affiliations:** ^1^Clinic of Psychiatry, Social Psychiatry and Psychotherapy, Hannover Medical School, Hannover, Germany; ^2^Institute for Biostatistics, Hannover Medical School, Hannover, Germany

**Keywords:** blinding, clinical trials, THC, RCT, cannabinoids, unblinding

## Abstract

Randomized double-blind placebo-controlled trials (RCTs) are regarded as the gold standard for clinical trials. While there are established standards to avoid unblinding, in RCTs using tetrahydrocannabinol (THC) containing cannabinoids, however, accidental unblinding and intentional self-unbinding must be considered as a particular issue, since THC tests are widely available. To investigate unblinding rates in an RCT using a THC-containing cannabinoid, we re-contacted 54 out of 97 participants of the CANNA-TICS trial who had participated in our study center in Hannover. Of the 54 participants, 53 could be reached. Of these, one participant (2%) stated that she had unblinded herself intentionally during the treatment phase, and another three patients (6%) reported intentional unblinding after the end of the treatment. Noteworthy, two patients provided discrepant information and denied self-unblinding during the interview, although during study/clinic visits they had reported having done so. Thus, based on all available information, three participants (6%) unblinded themselves intentionally during the treatment phase and another three (6%) after the end of the treatment. Accidental unblinding during the treatment phase was reported by 4/54 participants (7%) (during study visits). Since one participant reported both intentional self-unblinding (during the interview) and accidental unblinding (during a study visit), the total unblinding rate was 17% (*n* = 9). Of these, seven participants (13%) reported unblinding during the treatment phase. When asked in the interview whether they knew that self-unblinding would have been possible, only 34% (*n* = 18/53) of participants stated that they had been aware of this possibility. Thus, altogether 33% (*n* = 6/18) of those being informed about the possibility of self-unblinding did so and half of them (3/18, 17 %) during the treatment phase. It can be expected that in parallel to increasing knowledge of medicinal and recreational use of cannabinoids, more and more people will also be informed about the availability of THC tests. Hence, in future RCTs using THC-containing cannabinoids, researchers have to take the possibility of accidental and intentional unblinding into consideration, when designing the study.

## Introduction

Blinding is an important instrument to avoid bias in clinical trials (Noseworthy et al., 1994), since non-blinding and unblinding generally result in larger treatment effects (Schulz et al., 1995). Accordingly, double-blinding is regarded as the gold standard for clinical trials (Day and Altman, 2000). The term “double-blind” includes that during the study not only study participants are unaware of the assigned treatment, but also staff involved in study management, data collection, and data analysis. In general, it is easier to maintain blinding in pharmacological than non-pharmacological studies (Boutron et al., 2004). In pharmacological trials, there are established standards to avoid unblinding such as identical taste, smell, and appearance of the drug as well as the mode of delivery for each treatment group (Day and Altman, 2000). However, despite the best precautions, unblinding may occur due to different reasons.

In randomized double-blind placebo-controlled trials (RCTs) using cannabinoids, it has been speculated that specific treatment effects of cannabinoids might cause unblinding. However, in an analysis including three RCTs with a total of 666 patients with multiple sclerosis investigating the efficacy and safety of the cannabis extract nabiximols, it could be demonstrated that “typical” adverse events of cannabinoids did not lead to unblinding in treatment allocation (Wright et al., 2012). It has been suggested that in studies using nabiximols, blinding is effective because of slower onset of effect when using an oromucosal administration compared to inhalation and because of no or only mild psychoactive adverse events at clinically effective doses. In addition, peppermint flavor has been used for masking nabiximols' taste vs. placebo spray.

However, to the best of our knowledge so far, it has not been investigated whether intentional self-unblinding and accidental unblinding, for example, in the context of traffic checks or medical investigations are relevant problems in RCTs using tetrahydrocannabinol (THC) containing cannabinoids. While RCTs investigate the effects of newly developed drugs, these drugs are not available on the market and contain chemical ingredients generally unknown to the public; in contrast, THC-containing substances are widely available for medicinal and recreational use, and the use of THC can easily be proven by widely available THC tests.

Only recently, our group published the protocol of the RCT CANNA-TICS that aims to investigate the efficacy and safety of nabiximols in the treatment of adults with chronic tic disorders and Tourette syndrome (TS) (Jakubovski et al., 2020). Randomization of this study (*n* = 98) is already completed, and the publication of the results of treated patients (*n* = 97) is in preparation elsewhere. When designing the study, several efforts had been undertaken to guarantee blinding as best as possible such as (i) THC blood tests taken during the treatment phase were sent to a central laboratory of an unaffiliated institution to keep results confidential until the end of the study; (ii) in case of a traffic check, all participants were instructed to inform the policemen about participating in a study using a cannabis-based medicine; (iii) in case of medical investigations or emergencies (related or unrelated to study participation), participants were instructed to immediately inform medical staff not only about study participation, but also the possibility of unblinding, if THC blood or urine tests would be performed; (iv) information about the study protocol was sent to patients' general practitioners; (v) all participants received an ID card documenting participation in the CANNA-TICS trials including contact details of the study center; (vi) in case of unblinding, raters were changed, whenever possible; and finally (vii) for tic assessment, in addition to examiner and self-assessments, a video-based tic rating was used as a secondary outcome measure, and video assessments were done centrally by otherwise uninvolved blinded raters. However, we did not inform participants specifically about the possibility of unblinding by using a THC test, and participants were not specifically asked not to do so.

During a routine clinical visit at our TS outpatient department (independent from a CANNA-TICS study visit and after the patient had already completed all study visits), however, we were confronted with an unforeseen fact regarding the blinding of study participants: One of the study participants informed his treating physician (KMV) that he had unblinded himself during the course of the study by using a freely marketed THC urine test. Since this eventuality had not been taken into consideration, when designing the CANNA-TICS trial, we became interested in investigating unblinding rates in more detail and, in particular, to explore whether intentional self-unblinding is a general and so far neglected influential factor in “double-blinded” RCTs investigating the efficacy of THC-containing cannabinoids.

## Materials and methods

To identify how many study participants intentionally unblinded themselves during the course of the study by using widely available THC (urine) tests, we re-contacted all those study participants included at the study center at Hannover Medical School (MHH). CANNA-TICS was a multi-center RCT conducted in six study centers in Germany (*n* = 97 treated patients). Here, we decided to re-contact only study participants, who had been recruited at the study center at MHH, because (i) at MHH more than 50% of participants, who had been treated (54/97), had been recruited and (ii) we expected more truthful answers about adherence when being interviewed by already known study staff.

Between 3rd May and 3rd June 2021, we re-contacted all study participants recruited at MHH via phone using a structured interview to avoid any additional bias. All interviews were performed by a study assistant (MLZ) working at our center, who was not involved in CANNA-TICS assessments. We informed participants that (i) at the time of the interview, the CANNA-TICS RCT was still blinded, (ii) participation in the interview is voluntary, and (iii) additional questions are intended to receive information about study blinding. In addition to questions related to study blinding, we asked patients (i) how they did learn about the study, (ii) which study arm they do think they were assigned to, (iii) whether and how they benefitted retrospectively from the study medication, and (iv) whether they would decide again for participation in the CANNA-TICS trial. Finally, we asked participants for truthful answers, even if this would reveal non-compliance during the study. We assured that responses would have no negative consequences in any case.

In addition, we analyzed data on accidental and intentional unblinding from patients recruited at MHH obtained during and after the CANNA-TICS trial at both study and routine clinic visits in our center. The CANNA-TICS study protocol was approved by the responsible ethics committees (MHH No. 7386M, Eudra-CT-No. 2016-000564-42, ClinicalTrials.gov Identifier: NCT03087201) and the federal authorities.

## Results

Of the 54 study participants [mean age (+SD) = 37.4 years + 14.3, 40 men (74%)], 53 could be re-contacted [mean age (+SD) = 37.5 years + 14.5, 39 men (74%)]; all of them agreed to participate in the interview. Only one male participant was lost to follow-up.

During the interview, 18/53 (34%) participants stated that they knew that theoretically, self-unblinding would have been possible. Eight (15%) participants declared that they had thought about testing themselves for THC to figure out treatment allocation. One (2%) woman indicated that she intentionally unblinded herself two times during the treatment phase by performing a THC urine test. Another three (6%) participants (two men) indicated that they had used THC urine tests once to unblind themselves only after the end of the treatment. All four of these patients declared that they believed that they had received nabiximols.

Noteworthily, two study participants (two men) denied self-unblinding during the interview when being re-contacted after the end of the study, although they had indicated self-unblinding already at study or clinic visits during the course of the study. Altogether, three participants (two men) had informed their treating physician (KMV) (who had not been involved in clinical assessments of CANNA-TICS) about intentional self-unblinding. During regular study visits, five participants (four men) had informed study staff about intentional self-unblinding (*n* = 1) and accidental unblinding (*n* = 4, including the participant lost to follow-up), respectively (multiple answers possible). Participants reported the following reasons having caused accidental unblinding: (i) Two patients had been checked for THC during routine traffic controls without cause, although they had asked the policemen not to do so because of participating in a clinical trial, (ii) one patient had an emergency consultation unrelated to the CANNA-TICS trial that included a THC test, and (iii) one patient participated in a medical-psychological examination (MPU) that routinely includes drug tests.

Thus, based on all information available, three participants (6%) unblinded themselves intentionally during the treatment phase and another three (6%) after the end of the treatment. Four (7%) participants reported accidental unblinding. Since one participant reported both intentional self-unblinding (during the interview) and accidental unblinding (during a study visit), the total unblinding rate was 17% (*n* = 9). Of these, six participants (11%) were unblinded during the treatment phase (Table 1).

**Table 1:** Participants reported unblinding depending on the type of unblinding and study period.

**Type of unblinding**	**Time of reporting**	**Study period**	**Participants, n**
Intentionally	interview	treatment phase	1^#^
		after end of treatment	3^*^
	study visit	treatment phase	1
	clinical routine visit	treatment phase	2^#^
sum: intentionally unblinding			6^*^
accidentally	study/clinic visit	treatment phase	4^*^
total sum: unblinding			9^*^

* One participant reported intentionally self-unblinding after the end of the treatment during the phone interview and in addition accidental unblinding (police control) during the treatment phase during a study visit.

# One participant reported intentionally self-unblinding during both the phone interview and a clinical routine visit.

## Discussion

Clinician scientists, who design and conduct RCTs using THC-containing cannabinoids, should be aware that different from other blinded pharmacological studies, self-unblinding of participants is easily possible and cannot be completely avoided, since THC (urine) tests are widely available. Nevertheless, based on our data, it can be assumed that the adherence of study participants is relatively good, despite the theoretical option of unblinding. In our recently conducted CANNA-TICS trial (Jakubovski et al., 2020), 6% of study participants at MHH (3/54 participants) intentionally unblinded themselves during the treatment phase of the study, and another 6% (3/54 participants) after the end of the treatment. Five of these six participants believed that they had been given nabiximols.

However, only about one-third of participants (34%) had been aware that unblinding is easily doable. Thus, of those participants, who knew that unblinding is possible, 33% actually did so during the course of the study: 17% during the blinded treatment phase and another 17% after the end of the treatment. Since four participants (7%) had been unblinded accidentally during the treatment phase, altogether, seven participants (13%, one participant reported both intentional self-unblinding after the end of the treatment and accidental unblinding during the treatment phase) had been unblinded during the treatment phase.

CANNA-TICS investigated the effects of nabiximols in adults with chronic tic disorders and TS. This implies that mainly young men were included since tic disorders are three to four times more common in men compared to women and tics spontaneously decrease with age. In addition, a substantial number of patients already had self-treated with cannabis before entering the study. Finally, it can be assumed that mainly patients with general interest and at least some basic knowledge about cannabis-based medicines participated in CANNA-TICS. Thus, compared to other indications and study samples, it can be assumed that participants in the CANNA-TICS study might have been better informed about cannabis-based treatments in general and the availability of THC tests in particular.

In future studies using THC-containing cannabis-based medicines, researchers should consider the possibility of self-unblinding when designing the study. Although one might argue that informing study participants about the possibility of self-unblinding may result in an increased number of unblindings, we suggest addressing this topic proactively, when informing patients about the study. It can be assumed that the wide public will be increasingly well informed about medicinal and recreational use of cannabis including related issues such as car driving after the use of cannabis. Therefore, we do not expect that this approach will henceforth have the opposite effect by providing information on unblinding options. A possible approach would be to draw up a separate contract with study participants agreeing not to use THC urine or other tests for unblinding as suggested earlier in the context of the publication of study protocols before study completion (Basu et al., 2017). Alternatively, the possibility of self-unblinding could be addressed in the written informed consent that must be signed by all participants before entering the study. This seems more important since some participants provided conflicting information at different time points. This was somewhat surprising to us since for interviews, we re-contacted only study participants from our own study center to increase adherence and we assured that responses would have no negative consequences even if non-compliance would be revealed. We can only speculate about the reasons for discrepant answers. Patients may simply have forgotten that they had already indicated self-unblinding previously. Alternatively, patients might have been ashamed realizing through subsequent questioning that they had not behaved according to protocol.

Besides intentional unblinding, accidental unblinding occurred in 7% of study participants (*n* = 4/54), although several precautions had been undertaken including ID cards, information of the treating physician, and information on how study participants should react in case of traffic checks, medical investigations, and emergencies. Our data illustrate that accidental unblinding cannot be prevented in any case in RCTs using THC-containing cannabinoids and underline the importance of careful planning.

## Data availability statement

The original contributions presented in the study are included in the article/supplementary material, further inquiries can be directed to the corresponding author/s.

## Ethics statement

The study was approved by the Ethics Committee of Hannover Medical School, Hannover, Germany (MHH No. 7386M, Eudra-CT-No. 2016-000564-42, ClinicalTrials.gov Identifier: NCT03087201). The patients/participants provided their written informed consent to participate in this study.

## Author contributions

KM-V, CF, MH, and AP contributed to the conception and design of the study. EJ, CF, ML-Z, MH, and AP contributed to the acquisition of data. KM-V wrote the first draft of the manuscript. KM-V, CF, AG, CK, AK, MH, and AP contributed to the data analysis and interpretation of data. All authors contributed to manuscript revision, read, and approved the submitted version.

## Funding

The CANNA-TICS trial was funded by the German Research Foundation DFG (MU 1527/2-1). GW pharmaceuticals Ltd., provided nabiximols and placebos for this study.

## Conflict of interest

Author KM-V has received financial or material research support from EU (FP7-HEALTH-2011 No. 278367, FP7-PEOPLE-2012-ITN No. 316978), DFG: GZ MU 1527/3-1 and GZ MU 1527/3-2, BMBF: 01KG1421, National Institute of Mental Health (NIMH), Tourette Gesellschaft Deutschland e.V., Else-Kröner-Fresenius-Stiftung, GW pharmaceuticals, Almirall Hermal GmbH, Abide Therapeutics, and Therapix Biosiences; has received consultant's honoraria from Abide Therapeutics, Boehringer Ingelheim International GmbH, Bionorica Ethics GmbH, CannaMedical Pharma GmbH, Canopy Grouth, Columbia Care, CTC Communications Corp., Demecan, Ethypharm GmbH, Eurox Deutschland GmbH, Global Praxis Group Limited, Lundbeck, MCI Germany, Neuraxpharm, Sanity Group, Stadapharm GmbH, Synendos Therapeutics AG, and Tilray; is an advisory/scientific board member for Alexion, CannaMedical Pharma GmbH, Bionorica Ethics GmbH, CannaXan GmbH, Canopy Growth, Columbia Care, Ethypharm GmbH, IMC Germany, Leafly Deutschland GmbH, Neuraxpharm, Sanity Group, Stadapharm GmbH, Synendos Therapeutics AG, Syqe Medical Ltd., Therapix Biosciences Ltd., Tilray, von Mende Marketing GmbH, Wayland Group, and Zambon; has received speaker's fees from Aphria Deutschland GmbH, Almirall, Camurus, Cogitando GmbH, Emalex, Eurox Deutschland GmbH, Ever pharma GmbH, Meinhardt Congress GmbH, PR Berater, Spectrum Therapeutics GmbH, Takeda GmbH, Tilray, and Wayland Group. She has received royalties from Deutsches Ärzteblatt, Der Neurologie und Psychiater, Elsevier, Medizinisch Wissenschaftliche Verlagsgesellschaft Berlin, and Kohlhammer, all of these financial supports took place outside of this study. The remaining authors declare that the research was conducted in the absence of any commercial or financial relationships that could be construed as a potential conflict of interest.

## Publisher's note

All claims expressed in this article are solely those of the authors and do not necessarily represent those of their affiliated organizations, or those of the publisher, the editors and the reviewers. Any product that may be evaluated in this article, or claim that may be made by its manufacturer, is not guaranteed or endorsed by the publisher.
